# Changes in Peak Flow Value during Immunotherapy Administration

**DOI:** 10.1155/2012/212867

**Published:** 2012-02-07

**Authors:** Diego Saporta

**Affiliations:** Associates in ENT & Allergy, PA 470 North Aveue, Elizabeth, NJ 07208, USA

## Abstract

Nasal allergies are prevalent affecting a large percentage of the population. Not only the upper respiratory tract but the whole body is involved. Allergies produce morbidity (and even occasional mortality) as they can lead to asthma development, and increased number of accidents. Immunotherapy results can be evaluated by following symptom scores, medication use, and objective measurements. Using a Peak Flow Meter (PFM) to evaluate immunotherapy results, it became evident that patients with and without asthma exhibited an improvement in the Peak Flow (PF) value, suggesting that lower airway involvement in allergic patients could be more prevalent than assumed. A consecutive chart review was performed including patients of any age with nasal allergies (with or without asthma) treated with immunotherapy for at least 6 months that had at least 2 complete evaluations. When immunotherapy was successful, most patients exhibited an increase in the PF value regardless of asthma status. A very significant finding was that most allergy sufferers may have lower airway inflammation. The use of the PF value to assess immunotherapy results and the potential failure to diagnose asthma in allergy sufferers are discussed. A better diagnosis of lower airway inflammation could be substantial in the management of these patients.

## 1. Introduction

Nasal allergies are common and their prevalence in industrialized societies appears to be increasing. While nasal allergies were rarely diagnosed in the 19th century, their occurrence markedly increased during the 20th century. For example, studies show the incidence of nasal allergies in parts of the United States at 10% in 1974, 20% in 1986, and 42% in 1994 [1]. Similar figures of up to 40% have been reported in other parts of the world too [2]. In addition, similar increases have occurred in conditions not usually associated with allergies but with clear allergic etiology, such as eczema and asthma. These figures indicate an explosive increase. It is possible that an increasingly polluted environment is a causative factor [1].

Asthma is a common chronic disorder of the airways characterized by an underlying inflammation that leads to bronchial hyperresponsiveness and recurring airflow obstruction. In some cases patients develop persistent changes in airway structure, including fibrosis, smooth muscle hypertrophy, and angiogenesis. In the United States asthma affects more than 22 million people. It is one of the most common chronic diseases of childhood, affecting more than six million children in the USA [3].

The occurrence of asthma also has increased. Hospitalization rates are higher among young children. Collectively, individuals with asthma account for more than 497,000 hospitalizations annually. The onset of asthma for most patients begins early in life with the pattern of disease persistence. Recognizable risk factors include atopic disease. Current asthma treatment with anti-inflammatory therapy does not appear to prevent progression of the underlying disease severity [3].

The allergic disease affects the whole body. Fatigue is one of the most common complaints of the allergy sufferer, [4] and nasal congestion is recorded as the most bothersome symptom of allergic rhinitis [5]. Nasal obstruction is often the most severe symptom in patients with nasal allergies and it can lead to the onset or worsening of obstructive sleep apnea (OSA) [6].

Sleep-related symptoms are extremely common in patients with allergic rhinitis. Sleep impairment associated with allergic rhinitis is likely a major contributor to the overall disease morbidity, health care costs, and loss of work productivity [7].

 Individuals with OSA are at an increased risk for motor vehicle accidents [8] and lack of sleep has been implicated in job related accidents as well. [9] In addition, there are patients where the influence of nasal obstruction in sleep disordered breathing is critical [10]. Nasal obstruction also is a contributing factor to development of dentofacial abnormalities in the developing child [11].

So it is clear that having allergies means dealing with more than just nasal congestion, rhinorrhea, and itchy eyes. Not only can quality of life be significantly impaired for those with allergies, but also this condition can be potentially life threatening given that a patient with allergies is susceptible to fatigue, sleep deprivation, a higher incidence of accidents, and lower airway inflammation with bronchoconstriction.

While medical management of nasal allergies will control symptoms in the best of circumstances, immunotherapy is the only treatment modality available that can potentially cure the allergic condition [12, 13]. Immunotherapy works by modifying the immunological response of the allergic individual through stimulating the production of IgG (usually known as a “blocking antibody”) and eliciting more complex changes in the activity of the T-cells [14], where T-cell tolerance is attained mainly by generation of allergen-specific Treg cells leading to suppressed T-cell proliferation and Th1 and Th2 cytokine responses against the allergen. This is accompanied by a significant increase in allergen-specific IgG_4_, IgG_1_, and IgA and a decrease in IgE in the late stage of the disease [13].

In the clinical setting immunotherapy treatment is usually evaluated by following changes in symptoms scores. Specifically, the patient rates his/her symptoms usually using a numerical scale, and the change of this value over time is followed to assess improvement or lack thereof. Even though this is a subjective tool, different symptoms scores have been scientifically validated as useful [15–17]. Some of the objective measurements of symptomatic improvement include use of acoustic rhinomanometry [18].

**Figure 1:**
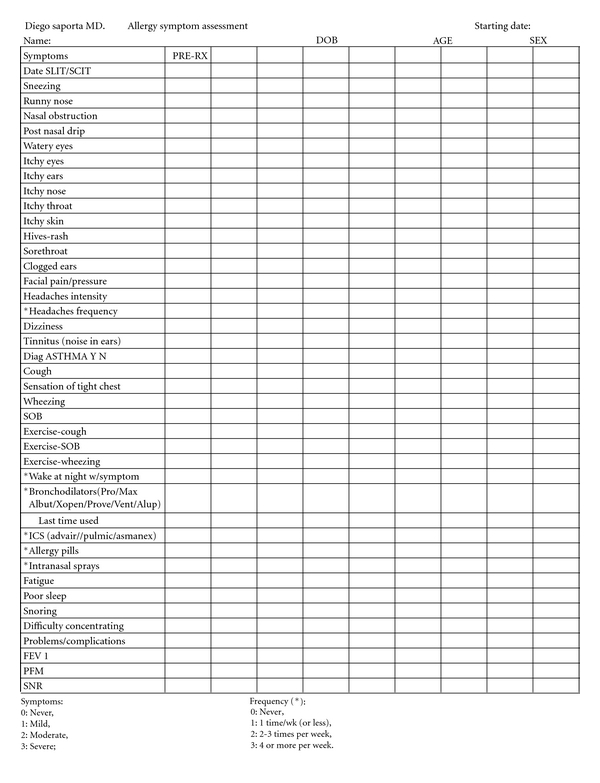


In our office we use a symptom questionnaire (see Figure 1) based on the scoring method followed by Fell [17]. We added objective measurements including nasal resistance determined by acoustic rhinomanometry and determination of PF value. While acoustic rhinomanometry requires significant training of the technician, it is time consuming, and yields results that are difficult to interpret, using a PFM device requires minimal training and yields easy-to-interpret results in seconds. It became clear that determining the PF value was a simpler procedure that could be easily incorporated in a private practice clinical setting. Soon after the use of the PFM was incorporated in our practice, it was observed that patients with allergies exhibited an improvement in PF value if immunotherapy was successful (as measured by a decrease in symptom scores and medication use) and that such improvement occurred not only in the patients with asthma but also in the allergy sufferer without any asthma symptoms.

To verify this hypothesis, we collected the data presented herein, with the understanding that this is only an observational study that lacks the rigor of a prospective randomized study with a control group.

## 2. Methods

A consecutive chart review was performed. Inclusion criteria for the study were patients of any age with nasal allergies (with or without asthma) treated with immunotherapy for at least six months that had at least two complete evaluations. A complete evaluation included symptom scoring, evaluation of medication use, and determination of PF value.

*Ethical Considerations*. Subjects' privacy was assured by the way data was collected and recorded so that subjects could not be directly or indirectly identified. In other words, a patient's privacy was protected by entering data for statistical analysis in a simple spreadsheet with nonspecific identifiers, such as patient number 1 and patient number 2, with subsequent refiling of the patient's chart according to usual procedure.

In our practice we follow Fell's method [17] for symptom scoring, which classifies each symptom with a numerical analog from 0 through 3 as follows:

0: symptom is not present,
1: symptom is mild,
2: symptom is moderate,
3: symptom is severe.

There were 21 symptoms considered for this evaluation (see Table 1). The symptom score was obtained by adding the values of each symptom (0–3) in each patient; the maximum total symptom score was 63. The total number of symptoms also was considered for monitoring clinical response.

When considering clinical response to immunotherapy, the use of medications is monitored as well. Patients that do not exhibit a decrease in medication use with the administration of immunotherapy are reassessed. For this evaluation we considered use of allergy pills (antihistamines or leukotriene receptor antagonists), intranasal topical steroids, and short acting bronchoagonists. No patient in this study was on oral or systemic steroids or decongestants.

For evaluation of medication use we use a similar numerical scale as follows:

0: medication is not being used,
1: medication is being used once a week or less,
2: medication is being used 2 to 3 times per week,
3: medication is being used 4 or more times per week.

The maximum total medication score was 9 and the maximum total number of medications was 3. When immunotherapy is successful, medication use will decrease regardless of the type of medication. In the asthmatic patient, asthma inhalers will be used less frequently or even discontinued entirely in the more successful outcomes.

The value of the PFM determination is used as the parameter to be recorded during each patient's visit.

The sample included adults and children with or without asthma treated with subcutaneous injection immunotherapy (SCIT) or sublingual immunotherapy (SLIT). Antigens were mixed according to results of an intradermal dilutional test. The data presented here was not analyzed according to test results. Patients on SCIT were treated according to American Academy of Otolaryngic Allergy (AAOA) guidelines [19]. Patients on SLIT were treated according to a previously published protocol [20]. The formulation of both injectable and oral allergy-vaccines was the same; based on allergy test results, all positive allergens were included in the treatment mixture.

Asthma symptoms considered included cough, sensation of tight chest, wheezing, shortness of breath (SOB), exercise-induced symptoms (cough, SOB, wheezing), or waking up at night with any of those symptoms. Presence of asthma symptoms in patients who considered themselves “nonasthmatic” was also analyzed.

Sixty charts that met inclusion conditions were identified. They were analyzed using the same criteria regardless if patients were treated with SCIT or SLIT. For each patient we evaluated total symptoms score, total number of symptoms, medication score, total number of medications used, and the PF value, which was obtained before treatment initiation and at least once more at the time of data collection.

The changes of the above parameters during the administration of immunotherapy were evaluated. In determining the results between two or more groups of patients, an ANOVA (Analysis of Variance) was performed using the PF performance as the dependent variable. When determining significance between continuous variables, a bivariate correlation analysis was performed.

## 3. Results

### 3.1. Demographics

The total sample included 60 subjects, ages 4 through 75 (mean 41.1 ± 17.5). Mean length of treatment was 22.9 ± 13.1 months. In 42 of 60 patients (70%), treatment extended for more than 12 months.

### 3.2. Asthma

**Figure 2:**
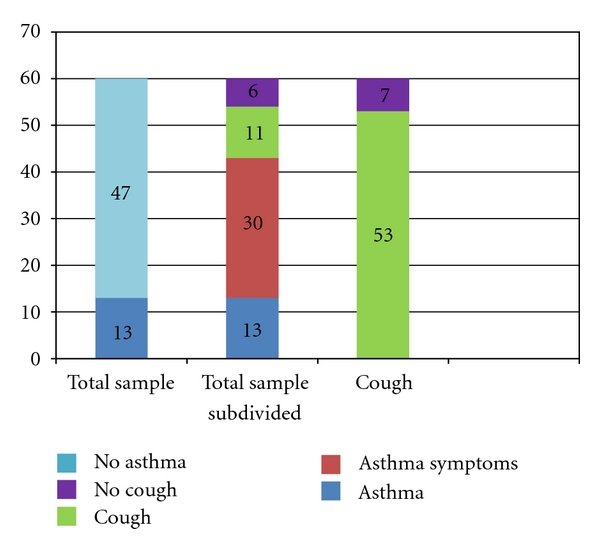
Total sample and subgroups. *Total sample*: 60 subjects, 13 report asthma, 47 do not. *Total sample subdivided*: 13 report asthma, Remainder 47: 30 have cough and other asthma symptoms, 11 have cough only, and 6 have no cough or other asthma symptoms. *Cough*: 53/60 subjects had cough.

A diagnosis of asthma was self-reported by 13 subjects (21% of the total sample), and only 1 of these subjects did not report cough as one of the symptoms. The remaining 47 subjects denied having asthma, although 30 reported asthma symptoms (30/47 = 63.8%) (see Figure 2).

The group of 47 patients that did not report asthma were divided into three subgroups (see Figure 2):

- patients with cough and other asthma symptoms: 30,
- patients with cough but no other asthma symptoms: 11,
- patients without cough (and no other asthma symptoms): 6.

### 3.3. Cough

Cough was reported by 53 out of 60 patients in this sample (88%). Eleven out of these 53 (subgroup b) had no other symptoms suggestive of lower airway inflammation. While it can be assumed that a patient with cough and other symptoms of lower airway inflammation has asthma, a patient with cough and no other symptoms is a different situation. Even though it is proven that cough can be the only symptom a patient with asthma may present, no further conclusions can be made without information on spirometric results or response to bronchodilator and/or anti-inflammatory therapy (see Figure 2).

Because all the information in this report comes from the symptom scoring sheet and not from office notes, we decided to consider the group “cough but no other asthma symptoms” as a separate group (see Discussion).

Adding the number of patients with asthma to the number of patients with cough and other asthma symptoms (13 + 30 = 43) suggests that 72% (43/60 = 71.6%) of the patients in a group of nonselected allergy patients are indeed asthmatic, and this is, at best, a conservative number as some of patients with “cough only” could also be asthmatic.

### 3.4. PF Changes

Average PF change for all 60 patients during immunotherapy increased from 376.23 (±115.01) at the beginning of treatment to 472.65 (±127.47) at the time of data collection for an overall improvement of 25.63%. From the total sample, 53 out of the 60 subjects (88.33%) showed an improvement in PF value, 4 had a worsening of PF value, and 3 had no change. Of the 53 subjects that showed an improvement in PF value, 48 had a decrease in symptoms score and number of symptoms. Therefore a PF increase has a predictor value of 90.57% for clinical improvement of the patient.

While an increase in the PF value is strongly associated with a symptomatic improvement (48/53), a decrease in the PF value is not necessarily associated with clinical worsening: of the 4 subjects with a worse PF value, all still had a decreased symptom score and 3 had a decrease in the number of symptoms. Of the 3 subjects with no change in the PF value, 2 got better (decrease in symptoms scores and number of symptoms) and 1 got worse.

### 3.5. PF Changes in Relation to the Number of Measurements

**Figure 3:**
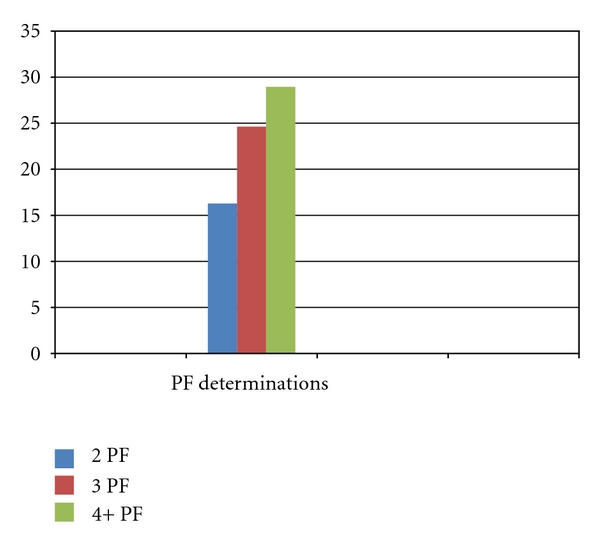
Percentage of PF value change in relation to the number of PF determinations. 2PF: two PF determinations. 3PF: three PF determinations. 4+PF: four or more PF determinations. The number of PF measurements is positively associated with the percentage of PF change (*r* = 0.357, *P* < 0.01).

In 11 patients there were 2 PF value measurements, one at the beginning of the treatment and the second one at the time of data collection (see Figure 3). Average PF change during immunotherapy for these patients ranged from 390.00 (±115.84) to 453.55 (±161.36), for an overall improvement of 16.29%.

In 13 patients there were 3 PF value measurements. Average PF change during immunotherapy for these patients ranged from 368.46 (±109.76) to 459.23 (±103.23), for an overall improvement of 24.63%.

In 36 patients there were 4 or more PF value measurements. Average PF change during immunotherapy for these patients spanned from 374.83 (±119.37) to 483.33 (±126.49), for an overall improvement of 28.95%.

A bivariate correlation analysis showed that the number of PF measurements is positively associated with the percentage of PF change (*r* = 0.357, *P* < 0.01). Therefore when more PF measurements are obtained, it is more likely to obtain a greater improvement in the PF value.

### 3.6. PF Value in Relation to Length of Treatment

There is a positive correlation between the change in the PF value and the number of months that the patient was treated (*r* = 0.253, *P* < 0.05). In other words, the longer a patient receives immunotherapy the more likely the PF value will increase.

### 3.7. PF Changes in Different Groups

**Figure 4:**
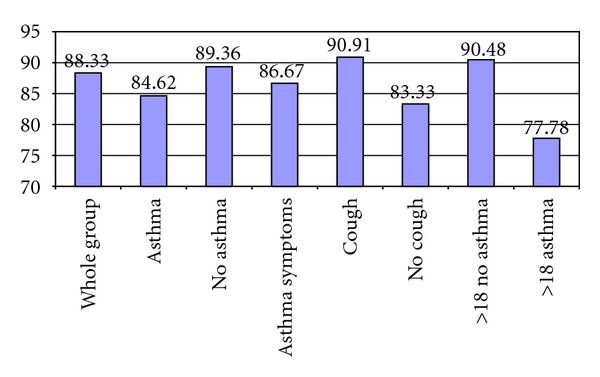
Number of subjects that had improved PF values in different groups. Whole group: 53/60; Asthma: Self-reported asthma patients: 11/13; No asthma: Patients that denied asthma: 42/47; Asthma Symptoms: Patients that denied asthma but had asthma symptoms: 26/30; Cough: Patients that denied asthma and had cough but no other asthma symptoms: 10/11; No cough: Patients that denied asthma and had NO cough and NO other asthma symptoms: 5/6. ANOVA showed no significant difference in average percentage of PF change of the aforementioned groups (*F* = 0.975, *P* = *N*/*S*). >18 No Asthma: Patients older than 18 years of age that did not report asthma: 38/42. >18 Asthma: Patients older than 18 years of age that reported asthma: 7/9.

PF changes were evaluated in patients that reported asthma and in the subgroups of the patients that did not report asthma (see Figure 4).

**Patients That Reported Asthma**

In the 13 patients who self-reported having asthma, the average PF value increased from 315.69 (±124.85) to 385.38 (±85.40), for an overall improvement of 18.08%. Eleven out of the 13 patients had an improvement in the PF value (84.62%). Two got worse (15.38%).

**Patients That Did Not Report Asthma**

In the group of 47 patients that did not report asthma, 42 (89.36%) had an improvement in the PF value, 3 did not improve (6.38%), and 2 decreased (4.26%). This group of 47 patients can be divided in 3 subgroups.

- For the thirty patients that did not report asthma but had asthma symptoms, the average PF value increased from 398.33 (±114.11) to 492.30 (±139.40), for an overall improvement of 23.59%. Twenty-six out of the 30 patients had an improvement in the PF value (86.67%). Three patients did not show any improvement (10.00%), and 1 got worse (3.33%).
- For the eleven patients that had cough but no other symptoms suggestive of lower airway inflammation, the average PF value increased from 376.36 (±109.39) to 510.91 (±105.49), for an overall improvement of 35.75%. Ten out of the 11 patients had an improvement in the PF value (90.91%). In 1 patient the PF value decreased (9.09%).
- For the six patients that had no cough or any other symptom suggestive of lower airway inflammation, the average PF value increased from 396.67 (±79.16) to 493.33 (±115.87) for an overall improvement of 24.37%. Five out of 6 patients had an improvement in the PF value (83.33%). In 1 patient the PF value decreased (16.67%).

An ANOVA showed that there is no significant difference among the average percentage of PF change of the aforementioned groups (*F* = 0.975, *P* = *N*/*S*).

### 3.8. Age

Because children can grow during a multiyear treatment (e.g., children between 3 and 13 years of age grow approximately 2 inches per year, and 2 inches can determine an increase of 15 to 25 points in PF value depending on the patient's sex), the same calculations were done excluding patients younger than 18 years of age. There were 51 patients older than 18 years of age.

The average PF value in these 51 patients (see Figure 4) changed from 395.76 (±110.01) to 495.49 (±120.97), for an overall improvement of 25.20%. In this group there were 9 patients (17.65%) that reported asthma and 42 patients (82.35%) that did not report asthma.

In the 42 patients that did not report asthma the PF value improved from 404.29 (±106.84) to 515.48 (±118.02), for an overall improvement of 27.50%. Thirty-eight out of the 42 showed an improvement in PF value (90.48%). The PF value did not change in 2 patients (4.76%) and decreased in 2 others (4.76%).

In the 9 patients that reported asthma, the PF value improved from 356.00 (±122.38) to 402.22 (±90.52), for an overall improvement of 12.98%. In 7 out of the 9 patients the PF value showed an improvement (77.78%). The PF value decreased in 2 (22.22%).

### 3.9. Pattern of PF Change

It was observed that patients could be divided into 2 groups according to the way the PF value changed during the administration of immunotherapy: in the first group the PF determination increased each time it was obtained, and in the second group the PF value fluctuated during the course of immunotherapy even though the value at the time of data collection was generally higher than the initial value.

Thirty-six patients (36/60: 60%) belonged to the first group (sustained improvement), and twenty-one patients (21/60: 35%) belonged to the second group (PF value cycled up and down). Three patients did not have any changes in the PF value (3/60: 5%).

In the first group 35 out of 36 patients (97%) had a decrease in the symptoms score, and 34 out of 36 patients (94%) had a decrease in the number of symptoms (coincident with an increase in the PF value).

For patients that consistently improved (35 of 60), the difference in symptom scores for those that had 5 or more PF value measurements was statistically higher than that for those that had fewer than 5 determinations (*F* = 5.02, *P* < 0.05). Number of symptoms also exhibited a similar pattern when comparing the same 2 groups (*F* = 6.42, *P* < 0.05).

## 4. Discussion

Patients with symptoms suggestive of lower airway inflammation may not consider themselves as asthmatic, but it is likely they are. A patient that only exhibits cough could be asthmatic, but unless an improvement in spirometric values after administration of bronchodilators is demonstrated or symptom improvement occurs after administration of asthma drugs, it is not possible to establish that patient as asthmatic.

Our sample consisted of 60 patients with allergies that were not screened for the presence of asthma. Of those patients, 13 self-reported having asthma and 30 reported experiencing asthma symptoms but did not report asthma, meaning that an impressive 71.67% of our sample potentially could be asthmatic. If the patients with cough but no other asthma symptoms were also considered, then this percentage could be even higher. If this finding is extrapolated to all allergy sufferers, it can then be assumed that the majority of allergy sufferers consulting an allergy practice might be asthmatic. It is clear that asking a patient if he/she is asthmatic is not sufficient; rather the presence of each symptom of asthma needs to be addressed. Patients with asthma are often at higher risk for severe reactions not only during the administration of immunotherapy [21, 22] but also during testing, and these patients should be considered more sensitive [23]. Fatalities from immunotherapy, although rare, are more common in asthmatics [24, 25]. Given all this, it is important to establish if an allergy patient is asthmatic or not, as a diagnosis of asthma affects quality of life as well as morbidity, and it can also potentially impact the life expectancy of that patient.

There is a tendency to consider asthma and allergic rhinitis (AR) as two separate entities, but there is strong evidence that this is not so. The term rhinobronchitis has been proposed to help recognize the concept of chronic inflammation throughout the entire airway in the patient with concurrent allergic rhinitis and asthma [26]. Up to 19% of hay fever sufferers develop asthma later in life [27]. Nasal challenge with environmental stimuli (such as cold air) leads to bronchoconstriction [26, 28]. Some AR patients with no perceived asthma develop bronchial hyperreactivity during AR exacerbation [26]. All this data supports the concept that the upper and lower airways are a unique entity impacted by a common, evolving inflammatory process. Therefore from an immunotherapeutic point of view, AR and asthma should be considered a single entity [29], and the results reported here support the concept of the Unified Airway [30]. If the allergic reaction influences the whole body, it is only logical that the bronchi will be involved in the widespread inflammation that affects the allergy sufferer.

There were 6 patients in the sample of 60 that were definitely not asthmatic. In these patients the average PF value improved 24.37%, and the PF value increased in 5 of 6 cases (83.33%). Both of these figures are in range with the other groups, which supports the idea that even nonasthmatic patients with nasal allergies still have lower airway inflammation. This explains why, even when a patient reports no asthma symptoms, the pulmonary function as assessed by the PF value improves with treatment. With this in mind, nasal allergies and asthma should be considered a continuum of the same disease that expresses itself differently in each patient; for certain individuals this means that the nose and the eyes will be more affected, and for others it means that the lower respiratory system will be more substantially impacted. However, it is a disease that at all times, even if to a minimal degree, probably involves all the organ systems.

This concept is difficult for patients to accept, and we find that when patients with hyperreactive airway are told they have asthma, the usual response is to deny the possibility. Still this is an important concept when treating patients with immunotherapy, since, as mentioned earlier, asthmatic patients are more likely to experience reactions [21, 22].

These results should contribute to raising awareness that potentially any patient presenting with allergy symptoms could also have lower airway hyperreactivity. As previously stated, the figure of 72% is possibly a conservative estimate; and if patients that had cough but no other asthma symptoms were considered as patients with airway hyperreactivity, the number could be even higher. In other words, in a sample of 60 nonselected allergy patients, it is possible that up to 90% ([13 + 30 + 11]/60) were asthmatic.

Having any symptoms suggestive of lower airway inflammation (and cough is one that is very frequently found) should lead to a work up to rule out the presence of a hyperreactive lower airway. We feel that if this was the case, the frequency of asthma-related diagnoses would markedly increase.

The fact that when more PF measurements are obtained the PF value will be higher could be related to the finding that the longer the number of months the patient is treated with immunotherapy the higher the PF value will be. This suggests that the longer the treatment, the better the results, regardless of the presence of asthma symptoms and age of the patient. In other words it appears that immunotherapy, when successful, leads to improvement of pulmonary function in all patients with allergies.

Immunotherapy is a treatment that can modify the immunological mechanisms that cause allergy symptoms [12]. It is an old treatment modality [31] that is proven to be effective [32–34]. With immunotherapy, it is expected that medication use will decrease, regardless of when the medication was started and which medication was used. When the treatment is fully successful, medications can be stopped. We only analyzed medication use, as the purpose of this study was not to address potential differences in effects of various medications, but to demonstrate that with immunotherapy medication use diminishes.

This is a retrospective study and, as such, it lacks the value of a prospective, randomized study with controls, which is very difficult to perform in private practice settings. The information reported in this study suggests that a PFM device could be useful in monitoring a patient's progress during immunotherapy, as changes in PF value can predict how a patient is doing from a clinical standpoint. The information it provides is available immediately, offering a quick assessment of patient's progress.

When PF value consistently improves, the predictor value of the PF change is very high. However, in a few cases these findings are relative: in some cases we observed that with an unchanged or decreased PF value, a patient can still experience some improvement (decreased symptom/medication score). We also found that with a better PF value, the patient can have clinical worsening (increased symptom/medication score). Overall, it appears that following a patient's PF value during immunotherapy is a good indicator of how he/she is responding to the treatment: a decrease in PF value can be used to predict that a patient is not doing well, and an increase in PF value can be used to predict that a patient is doing well, particularly if the increase in PF value is sustained over time.

We hope that this information will serve as a stimulus for authors in research facilities to plan a randomized prospective study with a control group where the usefulness of a PFM can be more properly evaluated.

## 5. Conclusion

Patients with nasal allergies that deny having asthma often have asthma symptoms. In our study, this occurred in at least 72% of the patients that denied having asthma. Therefore when taking a patient's history, it is critical to ask about the presence of each symptom of asthma, regardless of the patient's perception of asthmatic status.

If it is accepted that the allergic condition affects the whole organism, then it is only logical that the lower airway of an allergy sufferer will be involved. While the degree of involvement varies according to the individual, it appears from this data that the proportion of individuals with affectation of the lower airway is staggering. Perhaps then it is time to reassess the definition of asthma. A “looser” term such as lower airway inflammation or airway hyperreactivity would be more inclusive of all patients with asthma symptoms. Even the term rhinobronchitis [26] should be considered, as patients often reject the label of asthmatic. Regardless, it appears that lower airway inflammation is common and treatment of this inflammation either by anti-inflammatory inhaled corticosteroids, immunotherapy, or detoxifying interventions [35] could not only treat the present condition but perhaps, more importantly, prevent the development of irreversible lower airway remodeling.

Change in PF value can give a rapid assessment of how a patient is responding to treatment. When PF value increases, it is likely that a patient will improve in the majority of cases. In cases where the PF value is better in each determination, the PF has a 94% to 97% predictor value that the patient is doing well.

When PF value improves during the administration of immunotherapy, these changes are independent of asthmatic condition, the presence of any or no asthma symptoms, or the patient's age. Only length of treatment and number of PF measurements are related to an improved PF value.

## Figures and Tables

**Table 1:** Symptoms considered for scoring.

Sneezing
Runny nose
Nasal obstruction
Post nasal drip
Watery eyes
Itchy eyes
Itchy ears
Itchy nose
Itchy throat
Itchy skin
Clogged ears
Facial pain/pressure
Headaches
Cough
Sensation of tight chest
Wheezing
Shortness of breath
Exercise-induced SOB
Exercise-induced cough
Exercise-induced wheezing
Waking up with symptoms
